# Purification of a Hydrophobic Elastin-Like Protein Toward Scale-Suitable Production of Biomaterials

**DOI:** 10.3389/fbioe.2022.878838

**Published:** 2022-06-22

**Authors:** Sandra Haas, Monika Desombre, Frank Kirschhöfer, Matthias C. Huber, Stefan M. Schiller, Jürgen Hubbuch

**Affiliations:** ^1^ Institute of Process Engineering in Life Sciences, Section IV: Molecular Separation Engineering, Karlsruhe Institute of Technology (KIT), Karlsruhe, Germany; ^2^ Institute of Functional Interfaces, Karlsruhe Institute of Technology (KIT), Karlsruhe, Germany; ^3^ Center for Biosystems Analysis, Albert‐Ludwigs‐University Freiburg, Freiburg, Germany; ^4^ Cluster of Excellence livMatS @ FIT, Freiburg Center for Interactive Materials and Bioinspired Technologies, University of Freiburg, Freiburg, Germany

**Keywords:** thermoresponsive protein/polymer, protein purification, hydrophobic elastin-like protein (ELP), biomaterial production, process scalability

## Abstract

Elastin-like proteins (ELPs) are polypeptides with potential applications as renewable bio-based high-performance polymers, which undergo a stimulus-responsive reversible phase transition. The ELP investigated in this manuscript—ELP[V2Y-45]—promises fascinating mechanical properties in biomaterial applications. Purification process scalability and purification performance are important factors for the evaluation of potential industrial-scale production of ELPs. Salt-induced precipitation, inverse transition cycling (ITC), and immobilized metal ion affinity chromatography (IMAC) were assessed as purification protocols for a polyhistidine-tagged hydrophobic ELP showing low-temperature transition behavior. IMAC achieved a purity of 86% and the lowest nucleic acid contamination of all processes. Metal ion leakage did not propagate chemical modifications and could be successfully removed through size-exclusion chromatography. The simplest approach using a high-salt precipitation resulted in a 60% higher target molecule yield compared to both other approaches, with the drawback of a lower purity of 60% and higher nucleic acid contamination. An additional ITC purification led to the highest purity of 88% and high nucleic acid removal. However, expensive temperature-dependent centrifugation steps are required and aggregation effects even at low temperatures have to be considered for the investigated ELP. Therefore, ITC and IMAC are promising downstream processes for biomedical applications with scale-dependent economical costs to be considered, while salt-induced precipitation may be a fast and simple alternative for large-scale bio-based polymer production.

## 1 Introduction

In the recent years, the demand for high-performance products based on biological and renewable resources has risen (Thakur and Thakur, 2018; Muñoz-Bonilla et al., 2019). Nevertheless, the lack of simple production processes with affordable production costs is the reason for the current low market share of microbial polymers (Kreyenschulte et al., 2014). Maturation of recombinant DNA technologies allowed the development of engineered peptide-based polymers based on natural role models showing high resilience (Chow et al., 2008; Balu et al., 2014). In this context, elastin-like proteins (ELPs) are a class of genetically encoded biopolymers based on the repeating pentapeptide sequence Val-Pro-Gly-Xaa-Gly (VPGXG) found in the mammalian elastin with the guest residue Xaa representing any naturally occurring amino acid except proline (Urry, 1992; Rodríguez-Cabello et al., 2005). These engineered biopolymers show comparable mechanical properties as natural elastin, which enables their application in the biomedical and high-performance material field (Zhang et al., 2015). Several applications for ELPs are proposed, including ELP sequences as purification tags for different types of biomacromolecules (Meyer and Chilkoti, 1999; Meyer et al., 2001; Kim et al., 2007; Hu et al., 2010; Madan et al., 2013; Yeboah et al., 2016); ELPs as drug delivery systems, *de novo* organelles, and vesicular protocells (Simnick et al., 2011; Huber et al., 2015; Rodríguez-Cabello et al., 2016; Huber et al., 2019; Schreiber et al., 2019a; Schreiber et al., 2019b; Varanko et al., 2020); and ELPs as material for tissue repair or engineering (Caves et al., 2011; Varanko et al., 2020) or in 3D printing (Lin et al., 2019; Duarte Campos et al., 2020; Salinas-Fernández et al., 2020). ELPs with low transition temperature gained further interest as an interesting subgroup with potential future biomedical applications (Huber et al., 2014; MacEwan and Chilkoti, 2014; Bataille et al., 2016). Although several studies regarding the biosynthesis of ELPs have been conducted (Girotti et al., 2011), limited knowledge on the downstream processing of hydrophobic ELPs with low transition temperatures is available and, to the best of our knowledge, no industrial-scale production of any ELP exists.

Chromatography is a popular, scalable strategy for protein purification based on the interaction between ligands bound to a stationary phase and molecules in the mobile phase. In immobilized metal ion affinity chromatography (IMAC), this interaction is based on metal ions like nickel or cobalt, which show an affinity mainly for histidine and cysteine in aqueous solutions. To enhance the affinity of engineered genetically modified proteins, mostly polyhistidine tags are implemented into their amino acid sequence. Due to the high biospecific affinity of IMAC, a high separation efficiency is reached in a single-step purification, even under denaturing conditions such as high urea concentrations (Porath, 1992; Bornhorst and Falke, 2000; Gaberc-Porekar and Menart, 2001). The main drawbacks of IMAC are the need for a purification tag, hazardous consumables (typically, imidazole is used as competitive agent), and possible physiological and pathological effects of divalent cations on proteins (Gutiérrez et al., 2007; Tamás et al., 2014).

To overcome these drawbacks, (Meyer and Chilkoti, 1999) introduced inverse transition cycling (ITC) as a nonchromatographic purification route for ELPs or proteins with fused ELP tags. Below their transition temperature Tt, ELPs are soluble monomers showing full hydration. With rising temperature, polypeptide hydrophobicity is increased, their hydration is decreased, and molecular interactions between the ELP chains lead to aggregation of the now insoluble ELPs (Urry, 1992; Urry, 1997; Meyer and Chilkoti, 1999; Li et al., 2014). Other external stimuli, such as changes in ionic strength or pH, as well as protein concentration and protein characteristics such as molecular weight and the exact amino acid sequence, may induce this reversible precipitation (Urry, 1997). ELPs are most commonly expressed in *Escherichia coli* (*E. coli*) in order to obtain monodisperse polymers with precise control of their amino acid sequence. This allows transition temperature design with a precision of a few degree celsius, even when fused to another protein (Guda et al., 1995; Meyer and Chilkoti, 2002; Simnick et al., 2007; Chow et al., 2008).

As for microbial expression of ELPs in inclusion bodies (IBs)—such as for the ELP construct used in this study—these have to be solubilized before ITC purification. In general, IB solubilization is performed using high concentrations of chaotropic agents (e.g., guanidine hydrochloride or urea) or strong anionic tensides. These solubilization methods force protein unfolding, making a refolding of functional proteins into native conformation a crucial step toward their recovery for globular proteins (Burgess, 2009; Singh et al., 2015). Since ELPs are in a disordered state below Tt, no refolding step is required. Still, the weakening of hydrophobic interaction by chaotropic agents increases the Tt of the ELP (Zangi et al., 2009; Zhao et al., 2020). To avoid high centrifugation temperatures in the ITC process, buffer exchange against an aqueous buffer system without urea is usually performed by a salt-induced precipitation with the aim of ELP precipitation and subsequent dissolution in the desired buffer. This additionally proved to reduce the amount of host-cell protein (HCP) contamination (Hassouneh et al., 2010; MacEwan et al., 2014; Mills et al., 2019).

Purification by ITC starts with a sample containing soluble and insoluble impurities together with soluble ELPs and is based on repeating centrifugation steps below or above Tt. During the cold spin, the centrifugation step is performed at a temperature below Tt, separating insoluble contaminants from the soluble ELP. Vice versa, the ELP remains in the centrifugation pellet and is separated from soluble contaminants during the hot spin. The combination of a hot and cold spin is referred to as one cycle of ITC (McPherson et al., 1996; Meyer and Chilkoti, 1999; Hassouneh et al., 2010; MacEwan et al., 2014). As its main advantage, no specialized technical equipment or reagents are needed for an ITC purification (Trabbic-Carlson et al., 2004). However, the available industrial-scale centrifugation techniques are generally discontinuous, limiting the throughput and therefore hindering economic upscaling (Kohsakowski et al., 2020). Also, centrifugation steps are of high-energy consumption and therefore highly expensive, especially when heating or cooling steps are necessary (Nagarajan et al., 2013; Kwan et al., 2018).

To date, studies comparing the performance of purification processes of biomacromolecules TAGGED WITH either an ELP or polyhistidine showed a comparable product yield (Meyer et al., 2001; Trabbic-Carlson et al., 2004; Hu et al., 2010). Additionally, new purification processes such as microfiltration of ELP fusion proteins (Ge et al., 2006) and organic extractions (Verheul et al., 2018; Mills et al., 2019) were introduced. Still, IMAC and ITC are common downstream processes for ELPs as purification target, which are possibly suitable for large-scale productions. However, these purification methods were not compared to each other using a hydrophobic, polyhistidine-tagged ELP with low transition temperature as target molecule, which excludes the influence of different expression rates related to the applied tag.

In this study, salt-induced precipitation, ITC, and IMAC were assessed to purify a C-terminal hexahistidine-tagged ELP from fermentation broth in laboratory scale. The recently published hydrophobic construct ELP[V2Y-45] (Huber et al., 2014)—the guest residues are valine and tyrosine in a 2:1 ratio and the pentapeptide structure is repeated 45 times—was therefore chosen as the purification target. This construct promises distinct biomaterial properties toward its mechanical behavior and biocompatibility; yet, due its low-temperature transition behavior, it is more complex and difficult to purify (Huber et al., 2014). First, a simple nonchromatographic process based on high-salt precipitation (HSP) without further purification steps was performed. Second, one cycle of ITC was conducted after the salt-induced precipitation*.* Third, IMAC was used to purify the ELP and the leaked nickel ions were traced. All processes were performed with the same fermentation batch, formulated into the same target buffer, and discussed with a view to their final yield, removal of nucleic acids, and salinity of the final buffer system with regard to possible further applications and suitability for large-scale purification processes.

## 2 Materials and Methods

### 2.1 Materials, Buffers, and Protein Expression System

**FIGURE 1:**
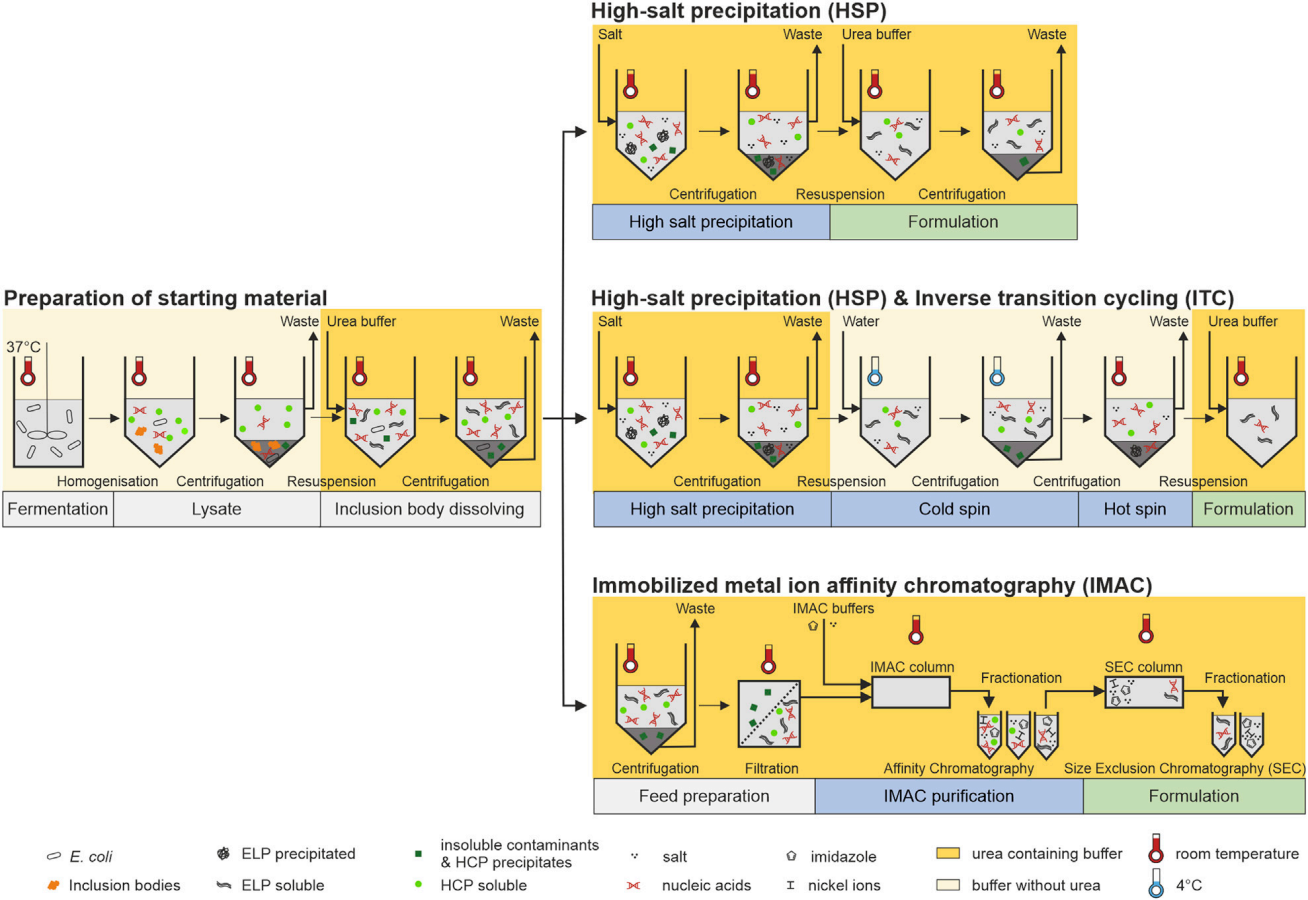
Schematic overview of the processes investigated in this study. Left: Fermentation, cell lysis, and dissolution of inclusion body were performed in one batch for all following processes to prepare the same starting material. Right-top: A high-salt precipitation and direct formulation by resuspension of the centrifugation pellet containing the target molecule in a urea-containing buffer system was performed as the most simple purification approach. Right-center: After the high-salt precipitation, one cycle of inverse transition cycling in a buffer system without urea was additionally performed starting with a cold spin below the transition temperature of the target molecule in ultrapure water followed by a hot spin above its transition temperature. Formulation of the target molecule was performed by a resuspension of the hot spin centrifugation pellet in a urea-containing buffer system. Right-bottom: Immobilized metal immobilized metal ion affinity chromatrography (IMAC) was used as an alternative purification approach. Prior to purification, the starting material was centrifuged and filtered to avoid column blockage. Formulation with the aim to reduce metal ion and salt ion contamination was carried out using a follow-up size-exclusion chromatography step.

The solutions and buffers were prepared with ultrapure water (PURELAB Ultra, ELGA LabWater, Lane End, United Kingdom). All buffers were pH-adjusted with 4 M sodium hydroxide solution and filtered through a 0.45-µm cellulose acetate membrane (Pall Corporation, New York, NY, United States) prior to usage. All chemicals were purchased from Merck KGaA (Darmstadt, DE). The investigated ELP is referred to as ELP[V2Y-45] following the nomenclature introduced by Meyer et al. (2001). The guest residues are valine and tyrosine in a 2:1 ratio and the pentapeptide structure is repeated 45 times. The one-vector toolbox platform approach developed by Huber et al. (2014) was used for plasmid generation. ELP[V2Y-45] with an expected molecular weight of 21.6 kDa due to its amino acid sequence (Supplementary Information S1) was expressed in IBs in *E .coli* strain Tuner (DE3) through isopropyl-β-d-thiogalactoside induction (Figure 1, *Fermentation*).

### 2.2 Preparation of Starting Material

With the obtained fermentation harvest, cell lysis was performed in three homogenization cycles using the high-pressure homogenizer EmulsiFlex-C3 (Avestin Inc., Ottawa, CAN) with a pressure of 1,000–1,500 bar. The IBs were dissolved either 1) during homogenization using a 20-mM sodium phosphate buffer (Na-PB) pH 8 containing 4 M urea followed by a centrifugation (30 min, 25°C, 17,000 g) or 2) by a separated IB dissolving step. Thereby, homogenization was performed in 20 mM Na-PB pH 8, and the cell lysate was centrifuged (30 min, 17,000 g) at 4 or 25°C. The resulting pellet was solved in 20 mM Na-PB pH 8 containing 4 M urea in a ratio of 5 ml/g for at least 1 h following a second centrifugation step (30 min, 25°C, 17,000 g). The resulting supernatant of the cell lysate centrifugation at 25°C was stored in aliquots at −20°C until further processing and is referred to as starting material in the following (Figure 1, Preparation of starting material).

### 2.3 High-Salt Precipitation

Ammonium sulfate (up to 1 M) and sodium chloride (NaCl, up to 1.5 M) were used to precipitate ELP[V2Y-45]—initially purified with two cycles of ITC—to determine the salt concentration needed for the precipitation of the target molecule. Therefore, protein concentration in the supernatant before and after centrifugation (20 min, 25°C, 17,000 g) was determined.

In the following process steps, a final concentration of 0.4 M ammonium sulfate (AMS) was dissolved in the starting material (Figure 1, *High-salt precipitation*). The solution was centrifuged (20 min, 25°C, 17,000 g) and the supernatant was separated from the pellet. For further analysis, the pellet of the HSP was resuspended in 7.5 ml/g 20 mM Na-PB pH 8 containing 4 M urea at room temperature (RT) followed by another round of centrifugation (20 min, 25°C, 17,000 g).

### 2.4 Inverse Transition Cycling

A salt-induced precipitation of the starting material was performed as described earlier using 0.4 M AMS. The resulting centrifugation pellet after the HSP was suspended in 7.5 ml/g ultrapure water at 4°C. Cold spin (4°C, 17,000 g) was performed for 2, 5, 10, 20, and 30 min. For the subsequent comparison of the purification routes, a centrifugation time of 2 min was chosen. The resulting supernatant was separated from the pellet and incubated at RT for 30 min before hot spin execution (20 min, 25°C, 17,000 g). The target protein in the centrifugation pellet was suspended in 20 mM Na-PB pH 8 containing 4 M urea for further analysis (Figure 1, *Inverse transition cycling*).

### 2.5 Immobilized Ion Metal Affinity Chromatography

The starting material was centrifuged (10 min, 25°C, 17,000 g) and the resulting supernatant was filtered with a 0.45-µm cellulose acetate membrane (Pall Corporation) prior to chromatographic steps (Figure 1, *Immobilized ion metal affinity chromatography*). All preparative runs were conducted with an Äkta Pure 25 chromatography system controlled with Unicorn 6.4.1 SP2 (GE Healthcare, Chicago, IL, United States). A HisTrap HP column (GE Healthcare) using nickel ions as binding sites was equilibrated with binding buffer (20 mM sodium phosphate, 4 M urea, 0.5 M NaCl, 20 mM imidazole, pH 7.4). The protein solution was applied using the sample pump. After a washing step of 10 column volume (CV) with binding buffer, a single-step elution with 5 CV elution buffer (20 mM sodium phosphate, 4 M urea, 0.5 M NaCl, 0.5 M imidazole, pH 7.4) was performed and fractionated. The elution fractions including target protein were pooled according to the measured absorption at 280 nm. A HiTrap Desalting column (GE Healthcare) was used as size-exclusion chromatography (SEC) in flow-through mode toward 20 mM Na-PB pH 8 containing 4 M urea for further analysis.

### 2.6 Analytics

Different quantitative analytical methods were assessed to determine the ELP[V2Y-45] concentration after each process step. Since the investigated ELP precipitated at RT in aqueous solutions, either the cooling of the whole analytical process was inevitable or the Tt had to be raised, for example, by addition of 4 M urea. However, these requirements did not allow the quantification of ELP[V2Y-45] containing process-related impurities with the assessed methods (see Supplementary Information S2).

#### 2.6.1 Purification Analytics

Sodium dodecyl sulfate polyacrylamide gel electrophoresis (SDS-PAGE) was used to evaluate the process step performance and the purity of the target molecule. Centriguation pellets were dissolved using the same buffer-to-pellet ratio as used for the corresponding process step. Samples were diluted by factor 100 (dissolved centrifugation pellets) or 65 (centrifugation supernatants) prior to analytics. SDS-PAGE was run on a PowerEase 500 Power Supply using NuPAGE™ running buffer, LDS sample buffer, and NuPAGE™ 4–12% BisTris protein gels (all Invitrogen™, Carlsbad, CA, United States), according to the manufacturer’s manual. Gels were stained with Coomassie^®^ brilliant blue G-250 solution and scanned with a Bio-5000 VIS Gel Scanner (Serva Electrophoresis, Heidelberg, DE). The target protein’s purity was determined by image processing of the SDS-PAGE gels using ImageJ V1.53e (NIH, Bethesda, MD, United States). Thereby, the ratio of the background-corrected integrated density of the target molecule band compared to the whole band was determined. The yield *Y* of the process was calculated by

$$Y=\;P_{ELP\left[V2Y-45\right]}*m_{protein,tot},$$ (1)

where $P_{ELP\left[V2Y-45\right]}$ is the purity of ELP[V2Y-45] determined by image processing of the SDS-PAGE gels as described and $m_{protein,\;tot}$ is the protein mass in the final formulation per ml of the starting material.

#### 2.6.2 Protein Quantification

Nucleic acid contamination was evaluated by the ratio of absorbance at 260–280 nm (*A*260/*A*280 ratio) and protein concentration of purified samples was measured *via* 280 nm absorbance using the spectrophotometer Nanodrop 2000c (Thermo Fisher Scientific, Waltham, MA, United States). The molar extinction coefficient at 280 nm *ε*_ELP[V2Y-45],280nm_ was determined to be 0.799 l/(g cm) in a concentration range up to 10 mg/ml in 20 mM Na-PB containing 4 M urea (Supplementary Information S3). For this, ELP[V2Y-45] was initially purified with two cycles of ITC, formulated in ultrapure water, and lyophilized for 72 h at 0.66 mbar and −55°C using the freeze-dryer Alpha1-4 LDplus (Martin Christ, Osterode am Harz, Germany). Conductivity measurements of formulated solutions with a concentration of 2 mg/ml ELP[V2Y-45] were performed to qualitatively compare the salinity of the final formulations using a conductivity meter CDM230 (Radiometer Analytical SAS, Lyon, FR).

#### 2.6.3 Molecular Weight Analysis

The molecular weight of purified ELP[V2Y-45] was evaluated for all proposed downstream processes by matrix-assisted laser desorption ionization-time of flight mass spectrometry (MALDI-ToF-MS) with an UltraFlextreme™ MALDI-ToF system controlled with the software FlexControl 3.4 (Bruker, Billerica, MA, United States). Each crystallized sample was manually targeted with the Smartbeam™-Laser (Bruker) at 355 nm. Formulated ELP[V2Y-45] was mixed in a 1:5 ratio with sinapinic acid and spotted on the MALDI target. All measurements were performed in a 10–50 kDa mass range using the Random Walk modus without automatic matrix suppression.

#### 2.6.4 Particle Size Analysis

Dynamic light scattering (DLS) measurements of the protein solutions were conducted with four replicates using the Zetasizer Nano ZSP (Malvern Panalytical, Malvern, United Kingdom). Each measurement was carried out with a sample volume of 50 µl in a quartz glass cuvette (ZEN 2112) and consisted of 20 acquisitions for 5 s per temperature step with a temperature ramp between 0 and 24°C in steps of 2°C. For these investigations, ELP[V2Y-45] was purified with two cycles of ITC, formulated in ultrapure water, and lyophilized for 72 h at 0.66 mbar and −55°C. Prior to measurement, ELP[V2Y-45] was dissolved in ultrapure water with a concentration of 2 mg/ml.

#### 2.6.5 Quantification of Leaked Nickel Ions

To quantify leaking metal ions at neutral pH in common buffer systems, a spectrometric assay using an UV/Vis spectrometer was used (Kokhan and Marzolf, 2019). The assay was performed as described by Swaim et al. (2019) with the difference that a 6% w/v hydroxynaphthol blue (HNB) stock solution was prepared. In short, the difference in the absorption spectra of the HNB dye and the formed HNB–metal complex at 647 nm is used to determine the metal ion concentration. A calibration line with nickel(II) chloride hexahydrate in a range from 0 to 4.5 µM in 0.45 µM steps was determined. The slope of the calibration was then used to calculate the nickel ion concentration in the calibration range for nonturbid samples. After a strip and recharge of the column, six method runs without protein load were conducted and the elution fractions as well as ELP[V2Y-45] containing solutions after the formulation with SEC were analyzed.

## 3 Results

### 3.1 Preparation of Starting Material

All purification routes were assessed using the same starting material which was prepared in one batch to eliminate possible preprocessing variances. As shown in the resulting SDS-PAGE (Supplementary Information S4), several proteins in the size range of 3.5–97 kDa as well as the target molecule—with an estimated size of 26 kDa in SDS-PAGE analytics—are present in the cell lysate and the starting material. In order to get an impression of the nucleic acid content during the purification process, *A*260/*A*280 ratios were determined to be 1.51 ± 0.01 for the cell lysate after homogenization, 1.53 ± 0.03 for the cell lysate pellet after centrifugation, and 1.71 ± 0.01 in the starting material after solubilization of the IBs (Supplementary Information S4; Supplementary Table S1). In order to ease handling during production, that is, by centrifugation at room temperature, the influence of centrifugation temperature after the dissolution of IB was assessed and evaluated by SDS-PAGE (Supplementary Information S5; Supplementary Figure S5A). Thereby, no difference in band intensities of a centrifugation temperature of 4°C compared to a centrifugation at 25°C could be observed by visual inspection. Additionally, direct dissolution of the IBs by performing cell lysis in a buffer system containing 4 M urea was conducted and evaluated by SDS-PAGE (Supplementary Information S5; Supplementary Figure S5B). In contrast to the previously described IB dissolution in a separated process step, the band intensity of the target molecule compared to the overall HCP band intensities is thereby less intense.

### 3.2 High-Salt Precipitation

**FIGURE 2:**
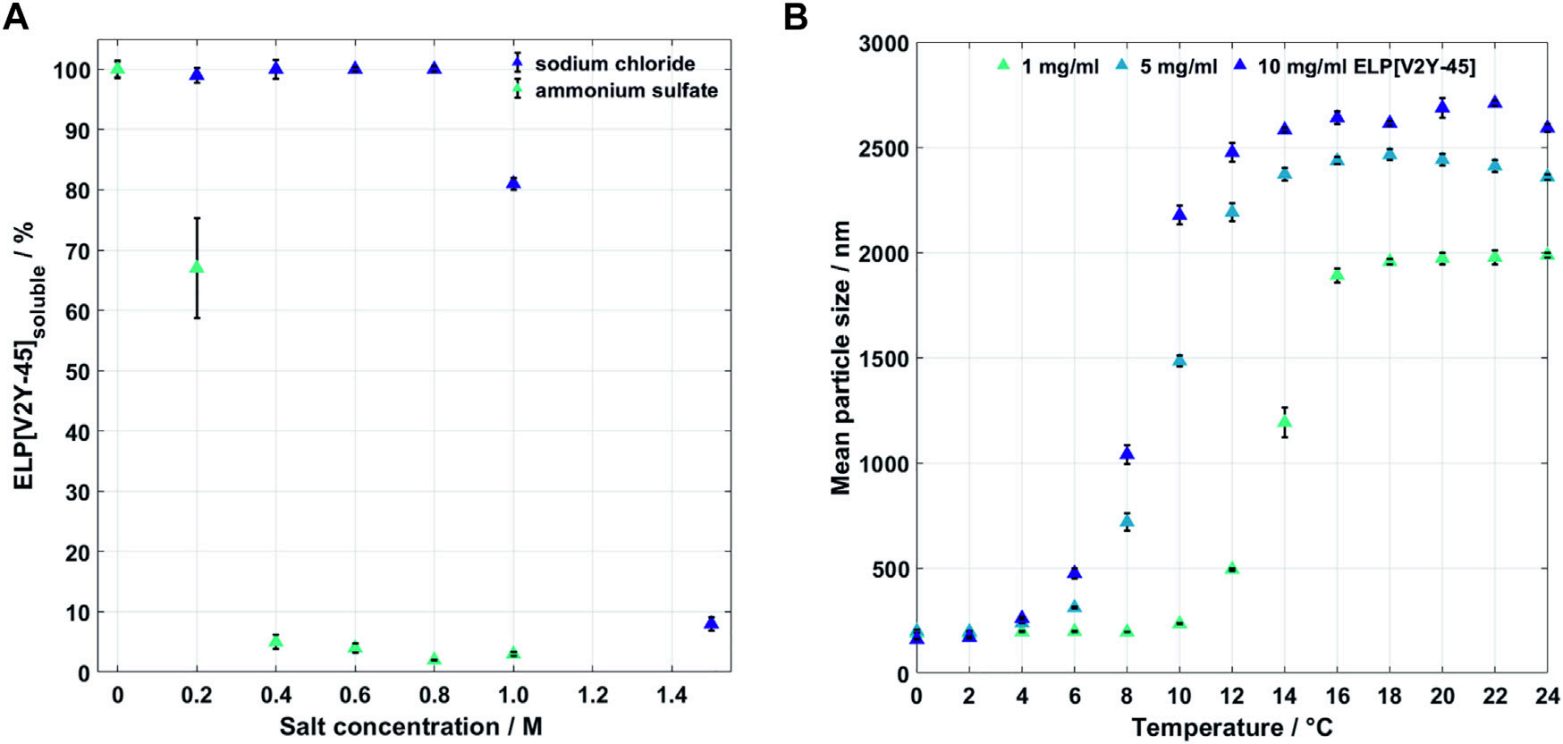
**(A)** Salt-induced precipitation of 1 mg/ml ELP[V2Y-15] in IB dissolving buffer containing 4 M urea at room temperature in IB dissolving buffer for sodium chloride (up to 1.5 M) and ammonium sulfate (up to 1 M) (*n* = 4). **(B)** Thermal dependency of the mean particle sizes for different concentrations of ELP[V2Y-45] in ultrapure water (*n* = 3).

The starting material was prepared in a buffer containing urea, in which no temperature-induced aggregation was observed up to 50°C, without higher temperatures tested (data not shown). Salt-induced precipitation, aimed at precipitating ELP[V2Y-45] in the IB solubilization buffer, was used to exchange the buffer toward an aqueous buffer without urea to allow ITC purification at moderate temperatures. To determine the required salt concentration, previously, ITC-purified ELP[V2Y-45] was precipitated with up to 1 M AMS and up to 1.5 M NaCl *via* two cycles (Figure 2A). A concentration of 0.4 M AMS precipitated 95% ± 1.2% of 1 mg/ml ELP[V2Y-45] and 1.5 M NaCl precipitated 92% ± 1.1%. Lower salt concentrations tested precipitated less than 50% of the target molecule for both salts. As for AMS, higher salt concentrations did not show to influence the precipitation ratio.

To verify whether both salts precipitated the target molecule in the presence of HCPs, 1.5 M NaCl and 0.4 M AMS were added to the starting material. Both salts showed to precipitate the target molecule (see Supplementary Information S6), whereby 0.4 M AMS was used for HSP and ITC purification routes in the further stages of the study.

**FIGURE 3:**
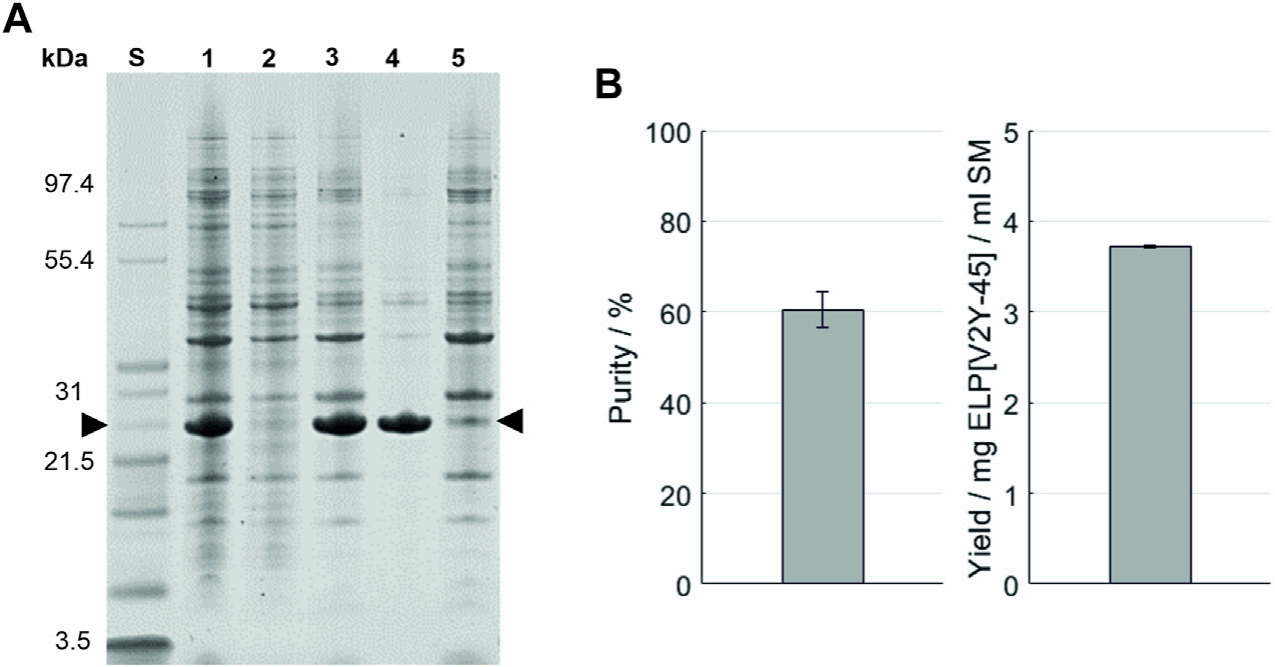
**(A)** SDS-PAGE analysis of the high-salt precipitation. An Invitrogen™ Mark12™ Unstained Standard (lane S) was used and the target molecule is indicated by arrows. Molecular weights of the selected proteins contained in the standard are shown on the left. The lanes are starting material (lane 1); supernatant (lane 2) and pellet (lane 3) after the high-salt precipitation; and supernatant (lane 4) and pellet (lane 5) after the centrifugation of the dissolved precipitation pellet. **(B)** Purity and target molecule yield per ml starting material were evaluated using image analysis of the SDS-PAGE gel.

As a first purification route, an HSP was performed with a subsequent resuspension of the precipitation pellet in the formulation buffer and analyzed *via* SDS-PAGE (Figure 3A). An overview of the buffer compositions of the different process steps of all purification routes can be found in the Supplementary Information S7. After the dissolution of IBs, HSP with 0.4 M AMS was conducted. The resulting supernatant and pellet as well as the pellet of the final formulation contain proteins in the entire size range analyzed (3.5–200 kDa) in SDS-PAGE. The target molecule’s band with a size of approximately 26 kDa (further indicated by arrows) is most prominent in the HSP pellet and consequently in the supernatant after centrifugation of the final formulation. In the final formulation—besides the target molecule—few protein bands with a size above 26 kDa are identifiable on visual inspection. Using this purification route, a yield of 3.72 ± 0.01 mg ELP[V2Y-45] per ml starting material with a molecular weight of 21,594.2 ± 0.8 Da was achieved as shown by MALDI-ToF analysis (Table 3). As determined *via* image analysis, the purity of the target molecule was 60.5% ± 3.9% (Figure 3B). The formulated solution showed a conductivity of 4.70 ± 0.04 mS/cm for a concentration of 2 mg/ml ELP[V2Y-45]. Nucleic acids remained predominantly in the HSP supernatant (*A*260/*A*280 = 1.94 ± 0.01), with the *A*260/*A*280 ratio of the HSP centrifugation pellet being 0.97 ± 0.04 and no further reduction during the formulation leading to a final *A2*60/*A*280 ratio of 0.99 ± 0.01 (see Supplementary Information S8).

### 3.3 Inverse Transition Cycling

As a second purification route, one cycle of ITC in ultrapure water was assessed. To gain an understanding of critical process parameters of the ELP[V2Y-45] phase transition, thermal transition behavior of ELP[V2Y-45] was initially investigated. The particle size of ELP[V2Y-45] in water depends on the solution temperature and protein concentration as shown in Figure 2B. For a protein concentration of 1 mg/ml, the mean particle size increases above a temperature of 10°C. Higher protein concentrations of 5 and 10 mg/ml already showed an increase in mean particle size for temperatures greater than 4°C. While the mean particle size increased, polydispersity was observed for all tested concentrations. This transition zone is smaller for 1 mg/ml, which showed monodispersity above 16°C compared to 14°C for both higher concentrations. Aggregation, indicated by a stable mean particle size, was reached for temperatures below RT for all tested concentrations.

**FIGURE 4:**
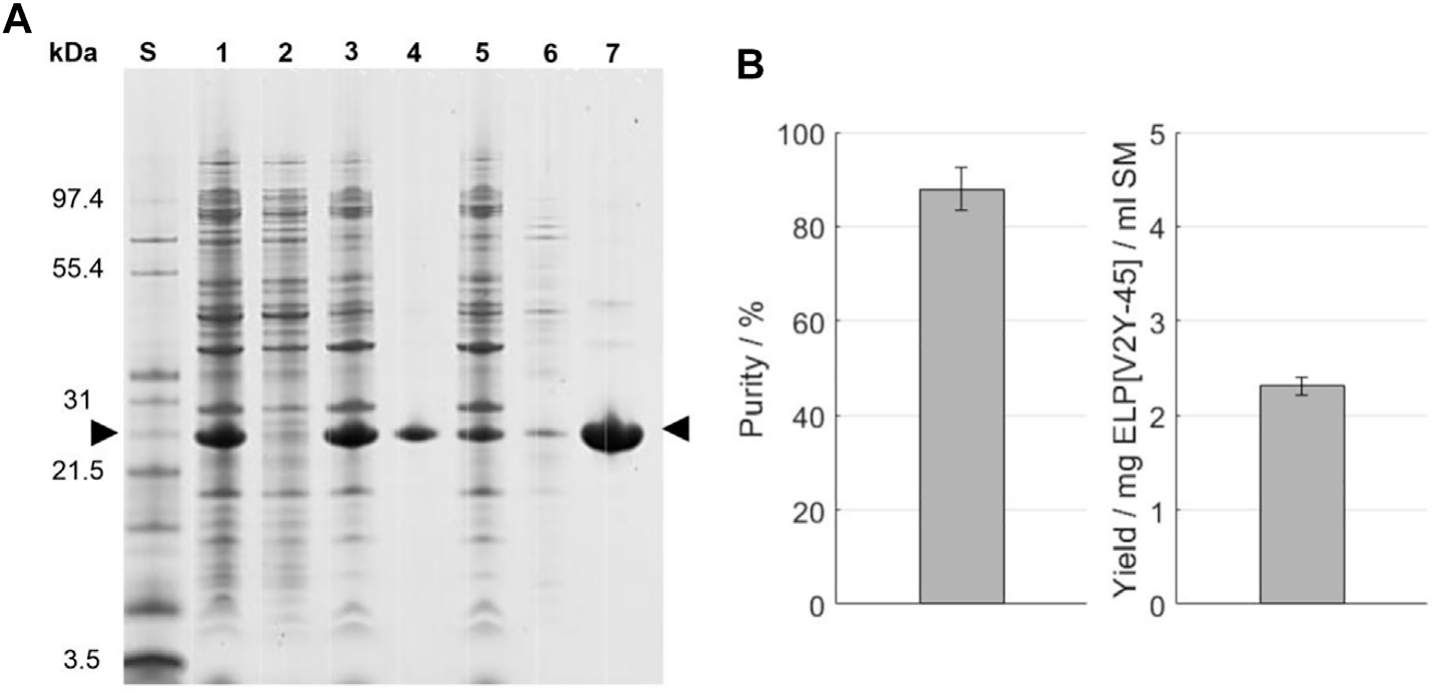
**(A)** SDS-PAGE analysis of the ITC purification. An Invitrogen™ Mark12™ Unstained Standard (lane S) was used and the target molecule is indicated by arrows. Molecular weights of the selected proteins contained in the standard are shown on the left. The lanes are starting material (lane 1); supernatant (lane 2) and pellet (lane 3) after the high-salt precipitation; supernatant (lane 4) and pellet (lane 5) after the cold spin; and supernatant (lane 6) and pellet (lane 7) after the high-temperature centrifugation of the cold spin supernatant (hot spin). **(B)** Purity and target molecule yield per ml starting material were evaluated using image analysis of the SDS-PAGE gel.

SDS-PAGE evaluation of one cycle of ITC in ultrapure water, which was applied after a salt-induced precipitation with 0.4 M AMS, is shown in Figure 4 A. The indicated target molecule band at a size around 26 kDa is predominantly visible in the starting material and HSP pellet. In the following cold spin process, the target molecule’s band shows a comparable intensity in the centrifugation pellet and supernatant, being less intense than in the HSP pellet. All other proteins show comparable band intensities in the cold spin centrifugation pellet compared to the HSP while being barely identifiable in the cold spin supernatant. The cold spin supernatant showed, with a *A*260/*A*280 ratio of 1.85 ± 0.07, a higher nucleic acid content compared to the cold spin pellet (0.86 ± 0.02) or the starting material (1.71 ± 0.01) (see Supplementary Information S8). After the hot spin, protein bands throughout the analyzed size spectrum which are less intense than in the cold spin pellet are identifiable in the centrifugation supernatant. The *A*260/*A*280 ratio in the hot spin pellet was reduced to 0.52 ± 0.00, while being 2.15 ± 0.01 in the hot spin supernatant. In the hot spin pellet, two protein bands in the size range between 31 and 55.4 kDa are visible to the eye. Compared to the target molecule band, these have a low intensity resulting in a target molecule purity of 88.0% ± 4.4% and a yield of the target molecule of 2.31 ± 0.09 mg ELP[V2Y-45] per ml starting material achieved as determined by image analysis (Figure 4B). The conductivity of the final formulation after dissolution of the hot spin centrifugation pellet was 3.62 mS/cm and the molecular weight determined to be 21,589.7 ± 1.1 Da. As evaluated by image analysis of the ITC-purification SDS-PAGE (Figure 4), a product loss of 17.05% ± 0.64% occurred during the cold spin centrifugation (Table 1). In order to maximize the yield of the target molecule, the influencing process parameters such as centrifugation time, target molecule concentration, and centrifugation temperature have been further evaluated. Longer centrifugation times of 30 min showed to double the target molecule’s loss up to 34.06% ± 0.92% as evaluated by image analysis of the resulting SDS-PAGE gel. A further decrease in centrifugation temperature to 2°C did not show an improvement in target molecule yield (data not shown). Also, increasing the buffer-to-pellet ratio to 10 ml/g for the HSP pellet and thus reducing the target molecule concentration during cold spin did not reduce the target molecule loss that occurred (data not shown).

**TABLE 1:** Centrifugation times between 2 and 30 min were tested for the cold spin conducted at 4°C. The purification performance of the cold spin was evaluated by the loss of the target molecule as determined *via* image analysis of SDS-PAGE gels (*n* = 3).

Centrifugation time	Loss of target molecule
(min)	(%)
30	34.06 ± 0.92
20	28.89 ± 4.24
10	23.39 ± 0.66
5	19.93 ± 4.45
2	17.05 ± 0.64

### 3.4 Immobilized Metal Affinity Chromatography

**FIGURE 5:**
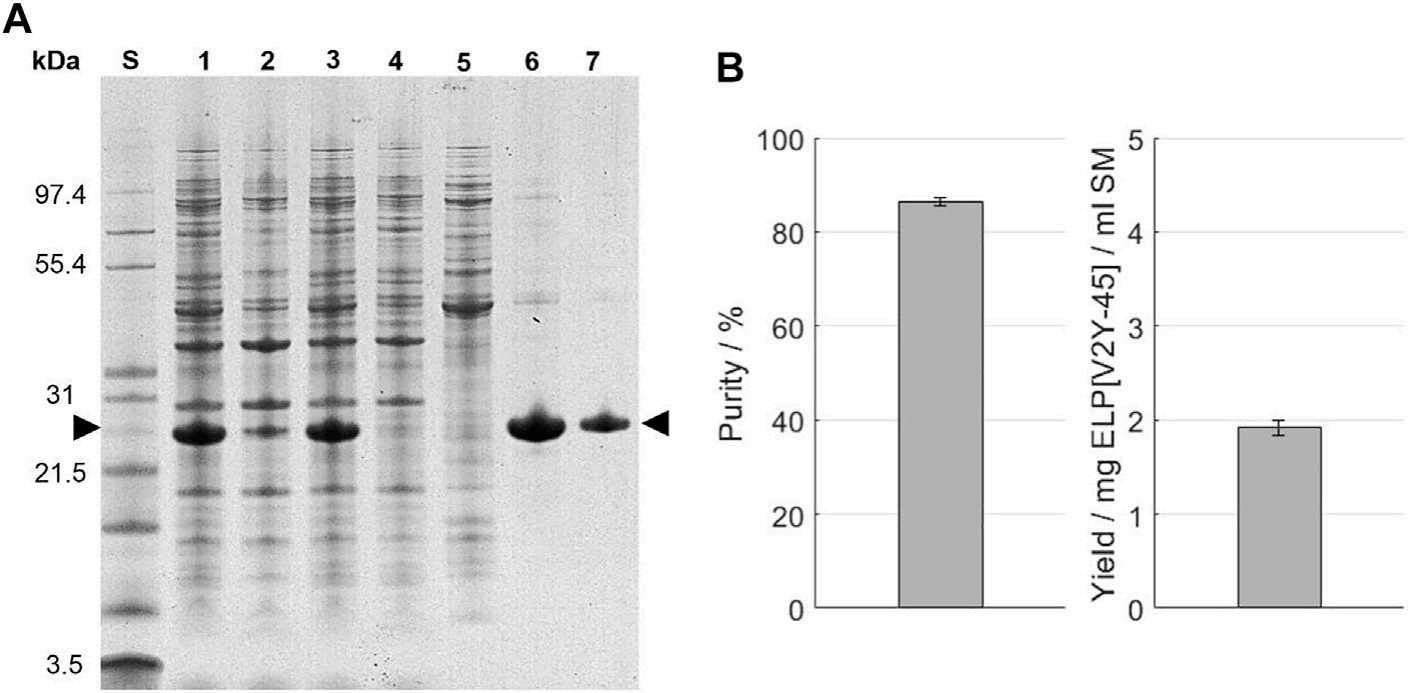
**(A)** SDS-PAGE analysis of the IMAC purification. An Invitrogen™ Mark12™ Unstained Standard (lane S) was used and the target molecule is indicated by arrows. Molecular weights of the selected proteins contained in the standard are shown on the left. The lanes are starting material (lane 1); IMAC feed (lane 3) and pellet (lane 2) after centrifugation; IMAC flow-through (lane 4); IMAC wash (lane 5); IMAC eluate (lane 6); and size-exclusion chromatography eluate (lane 7). **(B)** Purity and target molecule yield per ml starting material were evaluated using image analysis of the SDS-PAGE gel.

As a commonly applied purification method, immobilized metal affinity chromatography (IMAC) was performed for comparison. The chromatography feed was prepared by centrifugation and filtration of the thawed starting material. This led to a volume reduction of approximately 10% and a loss of the target protein of around 6% before sample loading on the column as evaluated by image analysis of the SDS-PAGE (Figure 5A). Protein bands are present throughout the analyzed size range in the starting material (*A*260/*A*280 = 1.71 ± 0.01), as well as the IMAC feed (*A*260/*A*280 = 1.75 ± 0.05) and the centrifugation pellet. A comparable band intensity for most protein bands except for the band of a protein size around 26 kDa and proteins with a size above 31 kDa is observable in the IMAC flow-through (*A*260/*A*280 = 1.82 ± 0.13). These bands are less intense in the flow-through compared to the IMAC feed solution. In the IMAC wash (*A*260/*A*280 ratio = 1.51 ± 0.01), protein bands are present throughout the analyzed size spectrum. Compared to the bands recognizable above 31 kDa, bands of smaller proteins are less intense. Addition of 20 mM imidazole could be used to reduce the amount of eluted proteins during the column wash, as indicated by a lower UV280 nm sum signal (Supplementary Information S9). In the IMAC eluate, the protein band with a size around 26 kDa is predominant, with less intense bands for proteins with a higher molecular weight being visible. After the formulation *via* SEC, the same protein bands than in the IMAC eluate are present in the SDS-PAGE, while being less intense. In the SEC, two separated peaks for the UV280 nm sum signal and conductivity signal were gained in the chromatogram, which were experimentally separated by fractionation (Supplementary Information S10).

The concentration of leaked nickel ions was determined using photometric assay and for consecutive chromatography method runs without the application of protein load. In the first method run after a column strip and recharge, the elution fraction contained 8.8 ± 1.6 µM nickel ions, while constantly decreasing to 4.2 ± 0.2 µM for the sixth consecutive run (Table 2). In the simultaneous presence of ELP[V2Y-45] and imidazole, the nickel assay became turbid and therefore the IMAC elution with protein loading was not analyzable (Supplementary Information S11). In the final formulation after SEC, no nickel ions could be traced and the molecular weight of the target molecule was 21,588.4 ± 0.1 Da. A purity of 86.5% ± 0.9% and a target molecule yield of 1.92 ± 0.08 mg per ml starting material was achieved (Figure 5B) with a conductivity of the final formulation of 3.59 ± 0.01 mS/cm and a *A*260/*A*280 ratio of 0.46 ± 0.00 (Table 3).

**TABLE 2:** Leaked nickel ion concentration depending on consecutive method runs without protein load after a strip and recharge of the IMAC column (*n* = 3).

Method runs	Ni^2+^ concentration
	(µM)
1	8.8 ± 1.6
2	8.0 ± 0.6
3	6.7 ± 0.2
4	5.3 ± 0.2
5	4.3 ± 0.2
6	4.2 ± 0.2

**TABLE 3:** Formulated ELP[V2Y-45] solutions of the three downstream processes were analyzed with regard to the target molecule’s molecular weight, its purity which was calculated from its band intensities in SDS-PAGE, the final target molecule yield, formulation buffer conductivity, and nucleic acid contamination in the final formulation (*n* = 3).

Process	Molecular weight	Purity	Yield	Conductivity	*A*260/*A*280
	(Da)	(%)	(mg ELP[V2Y-45]/ml starting material)	(mS/cm)	
HSP	21,594.2 ± 0.8	60.5 ± 3.9	3.72 ± 0.01	4.70 ± 0.04	0.99 ± 0.01
ITC	21,589.7 ± 1.1	88.0 ± 4.4	2.31 ± 0.09	3.62 ± 0.01	0.52 ± 0.01
IMAC	21,588.4 ± 0.1	86.5 ± 0.9	1.92 ± 0.08	3.59 ± 0.01	0.46 ± 0.00

## 4 Discussion

The aim of this study was to evaluate the purification performance of the three different processes with regard to their final yield, target molecule purity against HCPs and nucleic acids, as well as differences occurring in their final formulation and suitability for large-scale purification. Based on its amino acid sequence, the expected molecular weight of the investigated ELP[V2Y-45] is 21.6 kDa. As for SDS-PAGE analysis, the estimated size of the target molecule was around 26 kDa, which corresponds to a commonly described size overestimation by 20% on SDS-PAGE gels for other ELP constructs (McPherson et al., 1996; Meyer and Chilkoti, 2002). With direct IB solubilization during homogenization and homogenization in a nonsolubilizing buffer system with a subsequent solubilization, two different possibilities for IB solubilization were tested. Both approaches showed to dissolve IBs, while direct IB solubilization leads to a higher HCP contamination as indicated by SDS-PAGE analysis results. Therefore, a separation of homogenization and IB solubilization was performed for all the processes discussed. Typical lab protocols for ELP purification use low temperature during cell lysate centrifugation (Fong and Wood, 2010; Hassouneh et al., 2010; MacEwan et al., 2014). Increasing centrifugation temperature of the cell lysate after IB dissolution to 25°C did not show to influence the starting material composition compared to a centrifugation at 4°C for the investigated hydrophobic ELP[V2Y-45], which allows the reduction of cooling steps and therefore the overall processing costs. All purification routes were assessed with the same starting material with all process steps being carried out as one batch to avoid batch-to-batch variations.

### 4.1 High-Salt Precipitation

As typical for this class of genetically encoded proteins, ELP[V2Y-45] shows a transition behavior, which depends on the chemical composition and environmental stimuli. For efficient purification processes, profound knowledge of the temperature and salt-induced phase transition is beneficial. In the IB-dissolving buffer-containing urea, phase transition could not be induced up to at least 50°C. Urea is a well-known denaturant for proteins, which weakens the hydrophobic interactions and therefore raises the Tt without collapsing the ELP conformation (Zangi et al., 2009; Zhao et al., 2020). In order to counteract this shift, the addition of salts lowers the transition temperature and therefore precipitates the target molecule even for high urea concentrations present at RT, which then can be diluted in the desired target buffer system (Floss et al., 2010). Consistent with another study for ELPs, 1.5 M NaCl or concentrations greater than or equal 0.4 M AMS turned out to precipitate more than 90% of the target molecule (Fong et al., 2009). Higher NaCl concentrations were not tested, since such salt concentrations are highly corrosive and therefore undesired in the industrial scale. To reduce the overall salinity in the process, 0.4 M AMS was chosen for further experiments, albeit knowing that it precipitates more contaminant protein than sodium chloride (Fong et al., 2009; Floss et al., 2010).

The simple approach of an HSP followed by resuspension of the centrifugation pellet in the formulation buffer removed a major fraction of HCPs in a single step reaching a purity of 60.5 ± 3.9%. This is in a comparable range with the purification yield of 57 ± 2% shown for an ELP fusion protein earlier (Mills et al., 2019). Fewer process steps and a less complex sample handling—the entire process after IB dissolution is carried out in the same vial—are possible explanations for an increase in target molecule yield of 60.7% compared to ITC and 93.1% compared to IMAC. Also, nucleic acid contamination was reduced as indicated by a decrease in the *A*260/*A*280 ratio from 1.71 ± 0.01 to 0.99 ± 0.01. But, a slightly higher salinity is introduced into the final formulation as indicated by a higher conductivity compared to the more complex processes. Especially regarding possible applications which are sensitive to salt concentrations, such as hydrogel fabrication, this has to be taken into account (Mills et al., 2019). The main advantage of an HSP process is its simplicity, fast, efficient purification in a single step without temperature-dependent centrifugation steps or toxic consumables. For applications where higher purities, full removal of nucleic acids, or a control over the salinity is required, further processing would be necessary or other approaches should be preferred. Thus, for nonbiomedical applications—such as applications in environmental engineering (e.g., removal of heavy metals) (Kostal et al., 2003; Prabhukumar et al., 2004), bionanocomposite materials (Wang et al., 2011), or molecular sensors (Dhandhukia et al., 2013; Hassouneh et al., 2013)—the salt-induced precipitation process may be the best solution toward an industrial process scale for biomaterial production because of its simplicity and high target molecule yield.

### 4.2 Inverse Transition Cycling

For purification *via* ITC, the target molecule should be in a buffer system showing moderate transition temperatures, especially when working with ELP-tagged proteins, which may denature otherwise (Hassouneh et al., 2010). For the hydrophobic ELP[V2Y-45], this requires a buffer exchange toward an aqueous buffer without high salinity or high concentrations of chaotropic agents by an HSP with resuspension of the centrifugation pellet in ultrapure water. As described in common protocols, the cold spin was performed at 4°C (Hassouneh et al., 2010). For the investigated ELP, a loss of target molecule occurred in a range from 17% (2 min centrifugation time) up to 34% (30 min centrifugation time) in a single step. Although turbidity could not be determined by visual inspection, first agglomeration effects in ultrapure water could already be observed at 4°C using DLS for concentrations of 5 mg/ml and above. DLS is a commonly used technique for the determination of particle size distributions of nonturbid solutions (Ross Hallett, 1994; Hiroi and Shibayama, 2017), using the hydrodynamic radius of spherical particles for the size analysis of proteins. As ELPs show a disordered structure in soluble state and turbidity for temperatures above Tt, the calculated mean particle size very likely does not correspond to its actual geometrical size. However, using the same measurement setup, it is possible to assess relative dependencies and trends. Since protein concentration during the centrifugation step was above 10 mg/ml, first agglomeration effects have to be considered, which are further enhanced by longer processing times. Additionally, the centrifuge was heating up for long processing times at both tested centrifugation temperatures, which may have enhanced ELP[V2Y-45] precipitation. As higher protein concentration leads to a shift in the transition temperature toward a lower temperature (Urry, 1997; Despanie et al., 2016), a higher buffer-to-pellet ratio for resuspension of the HSP pellet was used to reduce the target molecule concentration during the centrifugation step. This, however, did not lead to an increase in the target molecule yield. This said, an even further decrease in protein concentration may be beneficial. On the other hand, further dilution leads to larger centrifugation volumes, which are unwanted in a large production scale.

ITC purification is a simple approach to further purify ELPs without specialized equipment in lab scale (Trabbic-Carlson et al., 2004; MacEwan and Chilkoti, 2014). During the hot spin, nucleic acid contamination could be further reduced compared to the HSP step and the highest purity (88.0% ± 4.4%) of all methods applied was achieved. Nevertheless, nucleic acids could not be reduced as efficiently for IMAC and may require additional processing for ITC-purified samples (Meyer and Chilkoti, 1999; Lim et al., 2007; Verheul et al., 2018). Still, highly efficient ITC purification is limited to ELPs without aggregation effects at low temperatures in relevant concentration ranges and buffer systems. An increase in the transition temperature by addition of chaotropic agents in the centrifugation buffer may increase the yield with possible simultaneous decrease in the target molecule’s purity. In this study, we focused on simple purification protocols with limited process steps to optimize the target molecule yield. However, more cycles would increase the purity at the cost of the overall yield. Still, the main drawback of this technique is the necessity of temperature-dependent centrifugation steps. Especially for large volumes, heating and cooling steps are time consuming and expensive, which may be a high barrier toward large-scale downstream processing using ITC.

### 4.3 Immobilized Metal Ion Affinity Chromatography

IMAC was used as a chromatographic approach for the purification of ELP[V2Y-45]. Elution of polyhistidine-tagged proteins is achieved using buffers that contain imidazole or other competing substances in addition to high-salt concentrations. The results of this study are comparable to the previously reported leached metal ion concentrations of <10 µM Ni^2+^ when using imidazole as eluant (Kokhan and Marzolf, 2019). Although protein functionality can be affected due to conformation changes after contact with divalent metal ions (Trabbic-Carlson et al., 2004), no evidence for chemical modification of ELP[V2Y-45] could be found in the molecular weight analysis. To reduce impurities from small molecules, such as metal or salt ions and imidazole, buffer exchange toward a target buffer system is required after IMAC. In this study, neither increased salinity compared to ITC nor metal ion contamination could be traced after subsequent preparative SEC. As described in the literature, IMAC proved to be a very efficient nucleic acid reduction (Murphy et al., 2003) with 0.46 ± 0.00 the lowest *A*260/*A*280 ratio of all processes discussed. Since no process step could be identified as the main cause for the loss of target molecule on the SDS-PAGE gels, we believe the product loss occurs due to a more complex sample handling. The additional centrifugation and filtration steps applied to avoid column blockage, as well as volume loss due to sample injection by sample pump, are possible sources for an overall material loss, which was not optimized in this study. Alternatively, HSP could be conducted instead of centrifugation and filtration as preprocessing steps before column loading. IMAC is a widely used process with a great wealth of experience in upscaling. A high purity after a single-step purification could be achieved in combination with the highest nucleic acid removal rate and low salinity after SEC. Still, the main drawbacks are high investment costs for necessary equipment such as chromatography resins, more complex sample handling compared to the other processes discussed, concerns regarding *in vivo* reactions due to the introduced tag, and the usage of toxic consumables thereby.

## 5 Conclusion

With salt-induced precipitation, ITC, and IMAC, three different processes were compared in regard to their purification performance for the hydrophobic ELP[V2Y-45] with low transition temperature. HSP without further processing leads to a 60% higher yield of target molecule. However, a lower purity of 60%, higher contamination with nucleic acids, and higher salinity have to be considered, which excluded, for instance, biomedical applications. When adding ITC to an HSP process, a purity of 88% after one cycle could be achieved. In contrast to common ELP constructs, the overall yield would be decreased by 17–34% during the cold spin at 4°C. Of course, more cycles could be conducted to reach higher purities at the cost of overall product yield and therefore were not further assessed in this study. It could be shown that affinity chromatography did not propagate molecular weight modifications of the target molecule. Further, metal and salt ions could be fully removed in a following SEC step. A comparable purity and a slightly lower yield compared to ITC were reached, which may be due to volume reduction related to a more complex sample handling. The effect of different expression rates based on a purification tag seems to be neglectable, since comparable target molecule purities and yields were shown for ELP fusions earlier. Both ITC and IMAC are therefore promising candidates for downstream processes with the aim of biomedical applications. However, further processing such as nuclease treatment or endotoxin removal might be necessary. A detailed consideration of processing costs for temperature-dependent centrifugation steps for ITC in contrast to high resin investment costs for IMAC has to be made for large-scale productions. For nonbiomedical applications without the need for high purities or nucleic acid reduction, a simple and fast salt-induced precipitation process may be the simplest solution in the ease of scalability and target molecule yield.

## Data Availability

The original contributions presented in the study are included in the article/Supplementary Material; further inquiries can be directed to the corresponding author.

## Abbreviations

*A*260/*A*280 ratio, absorbance ratio 260–280 nm; AMS, ammonium sulfate; CV, column volume; DLS, dynamic light scattering; ELP, elastin-like protein; HCP, host-cell protein; HNB, hydroxynaphthol blue; HSP, high-salt precipitation; IB, inclusion body; IMAC, immobilized metal ion affinity chromatography; ITC, inverse transition cycling; MALDI-ToF-MS, matrix-assisted laser desorption ionization-time of flight mass spectrometry; NaCl, sodium chloride; Na–PB, sodium phosphate buffer; RT, room temperature; SEC, size-exclusion chromatography; SDS-PAGE, sodium dodecyl sulfate polyacrylamide gel electrophoresis; *ε*_*i*,280 nm_, molar extinction coefficient of the protein *i* at 280 nm; $m_{protein,tot}$ , mass of all proteins in the target buffer solution; $P_{i}$ , purity of the target molecule *i*; Tt, transition temperature; and *Y*, process yield of the target molecule.
