# Supplementary material for: Purification of a Hydrophobic Elastin-Like Protein Toward Scale-Suitable Production of Biomaterials
Source: Front Bioeng Biotechnol. 2022 Jun 22;10:878838. doi: 10.3389/fbioe.2022.878838 (PMC9257828; doi:10.3389/fbioe.2022.878838)
Supplement: Supplementary file 1 [file DataSheet1.pdf]

Supplementary Material

Sandra Haas[1], Monika Desombre[1], Frank Kirschhöfer[2], Matthias C. Huber[3,4], Stefan M. Schiller[3,4], Jürgen Hubbuch[1,2]

[1] Institute of Process Engineering in Life Sciences, Section IV: Molecular Separation Engineering, Karlsruhe Institute of Technology (KIT), Fritz-Haber-Weg 2, 76131 Karlsruhe, Germany
[2] Institute of Functional Interfaces, Karlsruhe Institute of Technology (KIT), Hermann-von-Helmholtz-Platz 1, 76344 Eggenstein-Leopoldshafen, Germany
[3] Center for Biosystems Analysis, Albert-Ludwigs-University Freiburg, Habsburgerstr. 49, 79104 Freiburg, Germany
[4] Cluster of Excellence livMatS @ FIT, Freiburg Center for Interactive Materials and Bioinspired Technologies, University of Freiburg, Georges-Köhler-Allee 105, 79110 Freiburg, Germany

S1. Amino acid sequence of ELP[V2Y-45]

```
1        10        20        30        40        50
|        |         |         |         |         |
MDPMSSSGVPGVGVPGVGVPGYGVPGVGVPGVGVPGYGVPGVGVPGVGVP
GYGVPGVGVPGVGVPGYGVPGVGVPGVGVPGYGVPGVGVPGVGVPGYGVP
GVGVPGVGVPGYGVPGVGVPGVGVPGYGVPGVGVPGVGVPGYGVPGVGVP
GVGVPGYGVPGVGVPGVGVPGYGVPGVGVPGVGVPGYGVPGVGVPGVGVP
GYGVPGVGVPGVGVPGYGVPGVGVPGVGVPGYGGREASSHHHHHH
```

S2. Quantitative analytical methods tested

Cation exchange chromatography

Analytical cation exchange chromatography was conducted on a Vanquish Flex - R2L UHPLC System (Thermo Fisher Scientific) equipped with a TSKgel Q-STAT column (Tosoh Bioscience, Tokyo, JP). The column was equilibrated for 2 min with low salt buffer (10 mM citrate containing 4 M urea, pH 3) before a 10 µl sample injection. A column wash for one more minute with low salt buffer was performed before a 6 min linear gradient from 0 to 100 % with high salt buffer (10 mM citrate containing 4 M urea and 0.75 M sodium chloride, pH 3), which was hold for 4 minutes. Finally, the column was re-equilibrated with a 2 min wash using low salt buffer. All samples were prior filtered through 0.45 µm cellulose acetate filter (Pall Corporation, New York, US-NY). An initially via two cycles of ITC purified sample was applied to determine the ELP[V2Y-45] retention time, before process solution of the IMAC feed was loaded onto the column.
In order to evaluate the purity of the ELP[V2Y-45] elution peak, it was fractionated and prepared for matrix-assisted laser desorption ionization-time of flight mass spectrometry (MALDI-ToF-MS). Therefore, Vivaspin® ultrafiltration units (Sartorius Stedim Biotech, Göttingen, DE) with a molecular weight cut off of 10 kDa were used to reach a final concentration of at least 2 mg/ml total protein concentration. Meanwhile, a buffer exchange towards 10 mM ammonium bicarbonate was performed by three centrifugation cycles. Molecular weight analysis was performed as described in the main text.

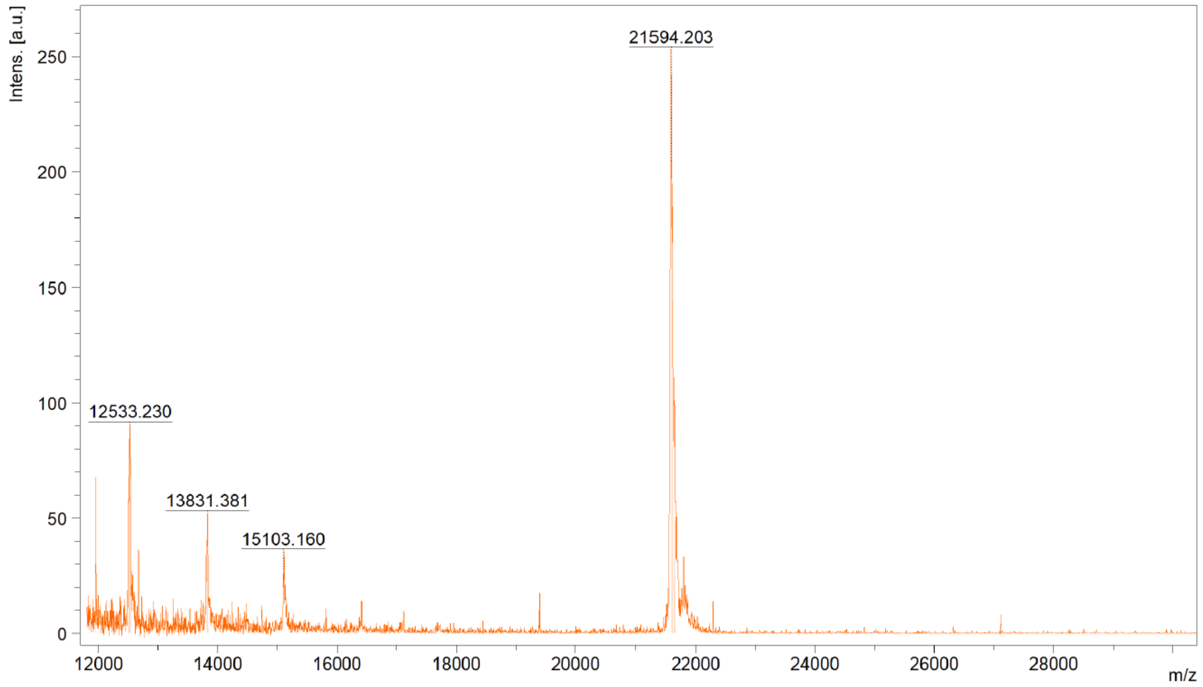

Figure S2 *- Intensities of the mass to charge ratio (m/z) of the fractionated, concentrated and rebuffered elution of the UHPLC elution peak containing ELP[V2Y-45]. Four peaks were identified by the spectrometer, three in a range below 16000 m/z and a main peak at 21594.203 m/z corresponding to ELP[V2Y-45].*

Besides ELP[V2Y-45], three more proteins with a size below 16 kDa could be traced in the elution peak (see Figure S2). Therefore, no quantitative analysis of the target molecule could be reached by this analytical method.

Size Exclusion chromatography

Size exclusion chromatography can be conducted using urea containing buffer systems. Here, initially via two cycles of ITC purified ELP[V2Y-45] as well as two model proteins BSA (Merck KGaA) and lysozyme from chicken egg white (Hampton Research, Aliso Viejo, US-CA) were used as references for host-cell protein (HCP) contamination. All proteins were separately prepared with a concentration of 2 mg/ml in a 20 mM Tris (Tris(hydroxymethyl)aminomethane, Merck KGaA) buffer pH 8 containing 4 M urea, which was also used as running buffer in the SEC method. A constant flow rate of 0.3 ml/min was applied to a Zenix SEC-300 column (Sepax Technologies, Newark, US-DE) which was implemented in a Vanquish Flex – R2L UHPLC system. Thereby, two peaks could be observed for ELP[V2Y-45]. The main peak after a retention time of 4.9 minutes covered 97.9 % of total area with a peak shoulder. A smaller peak (2.1 % of the total peak area) could be traced after 2.9 minutes. As one model protein, BSA showed multiple peak behavior and its signal overlapped with the ELP main peak. The second model protein lysozyme showed a pronounced peak tailing. Due to the unsharp ELP peak and difficult model protein behavior, this approach was not further assessed with process solution.

Reversed-phase chromatography (RP)

In preliminary experiments, ELP[V2Y-45] was dissolved with a concentration of 1 mg/ml in common RP running buffers. Thereby, it showed to be insoluble in isopropanol, hexane and acetonitrile without further addition of urea. Since RP separation is based on hydrophobicity and the presence of urea affects hydrophobic interactions no further tests were conducted.

Capillary electrophoresis

Capillary electrophoresis was conducted on a Caliper LabChip®GX II device (PerkinElmer, Waltham, US-MA) according to manufacturer`s protocol. Since ELP[V2Y-45] precipitated during sample preparation and no results could be obtained, sample preparation was adapted by dissolution of the analyzed sample in 20 mM Tris buffer pH 8 containing 4 M urea. As verification that this adaption does not influence analytical quality, lysozyme was used as a protein standard, which showed similar results for both sample preparation methods. However, for ELP[V2Y-45] which was previously purified via two cycles of ITC, estimated protein size was above 52 kDa and no defined single peak could be identified. For an HCP containing sample, HCP signal overlayed the fluorescence signal of the ELP[V2Y-45]. Therefore, this method was not applicable for quantitative or qualitative analysis.

S3. Extinction coefficient ELP[V2Y-45]

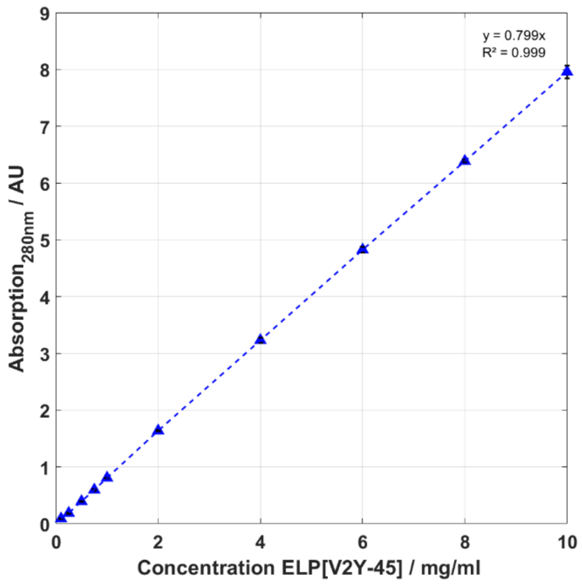

Figure S3 *- The extinction coefficient was determined performing an absorption measurement at 280nm using the NanoDrop2000c (Thermo Fisher Scientific, Waltham, US-MA) UV-Vis spectrophotometer. A dilution series between 0.1 to 10 mg/ml of lyophilized ELP[V2Y-45] was prepared in an aqueous buffer system containing 4 M Urea. According to Lambert-Beer law* $\varepsilon_{ELP[V2Y-45],280nm} = 0.799$ *L/(g*cm) in the linear absorption range up to at least 10mg/ml. (n = 3)*

S4. Preparation of starting material

Several proteins in the 3.5 to 97 kDa size range are present in the cell lysate. Beside other protein bands, a clearly pronounced band at approximately 26 kDa is observable in the lysate centrifugation pellet, while this band does not stand out in the lysate centrifugation supernatant. The addition of a buffer containing 4 M Urea to the resulting pellet solubilized the inclusion bodies containing the target molecule. After a following centrifugation, the band at 26 kDa in the supernatant is more clearly pronounced than in the centrifugation pellet, while other HCP bands are more pronounced in the pellet compared to the supernatant.

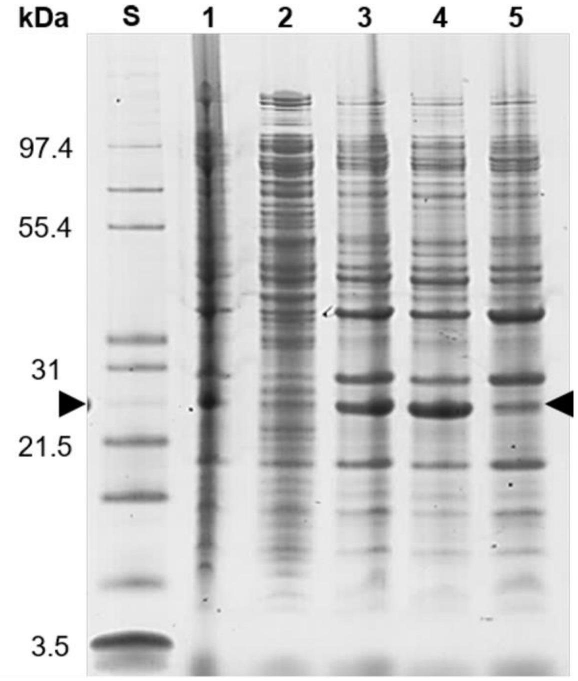

Figure S4 - *SDS-PAGE analysis of the start material generation. An Invitrogen™ Mark12™ Unstained Standard (lane S) was used and the target molecule is indicated by arrows. Molecular weights of selected proteins contained in the standard are shown on the left. The lanes are: cell lysate (lane 1); supernatant (lane 2) and pellet (lane 3) after the lysate centrifugation; the generated start material (lane 4) and centrifugation pellet (lane 5) after the inclusion body dissolving.*

Table S1 *– A260/A280 ratios during the preparation of the start material for all following process steps. The lysate in an aqueous buffer without urea was measured before and after centrifugation. The lysate pellet was resuspended in a buffer system containing* 4 M urea *for inclusion body dissolution and got centrifuged again (n = 3).*

**Sample**	**A260/A280**<br>-
Lysate - before centrifugation	1.51 ± 0.01
Lysate - centrifugation supernatant	1.66 ± 0.11
Lysate – centrifugation pellet	1.53 ± 0.03
Inclusion body dissolving – centrifugation pellet	1.32 ± 0.01
Inclusion body dissolving – centrifugation supernatant	1.71 ± 0.01

S5. Homogenization with and without urea

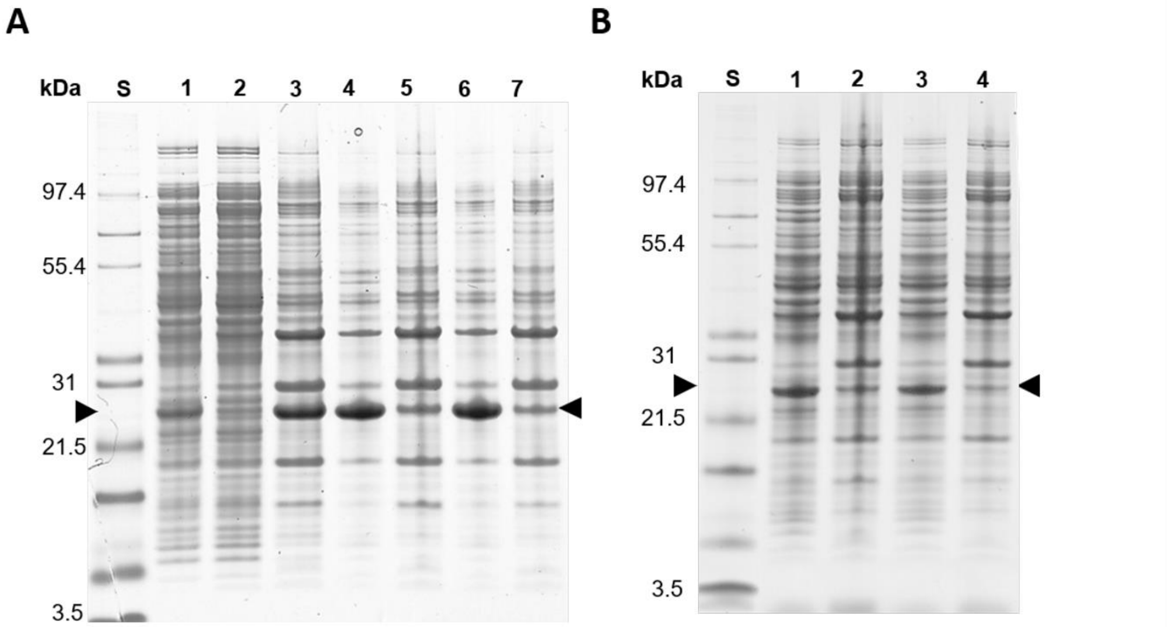

Figure S5 - *SDS-PAGE analysis of the homogenization without urea present during homogenization (A) and with urea present during homogenization (B). The lanes are: (A) cell lysate (lane 1); supernatant (lane 2) and pellet (lane 3) after the lysate centrifugation at 25°C; supernatant (lane 4) and pellet (lane 5) after centrifugation at 25°C of the dissoluted lysate pellet in urea-containing buffer; supernatant (lane 6) and pellet (lane 7) after centrifugation at 4°C of the dissoluted lysate pellet in urea-containing buffer; (B) supernatant (lane 1) and pellet (lane 2) after the lysate centrifugation at 25°C; supernatant (lane 3) and pellet (lane 4) after the lysate centrifugation at 4°C. An Invitrogen Mark 12 Unstained Standard (lane S) was used and the target molecule is indicated by arrows. Molecular weights of selected proteins contained in the standard are shown on the left.*

S6. Salt-induced precipitation of start-material

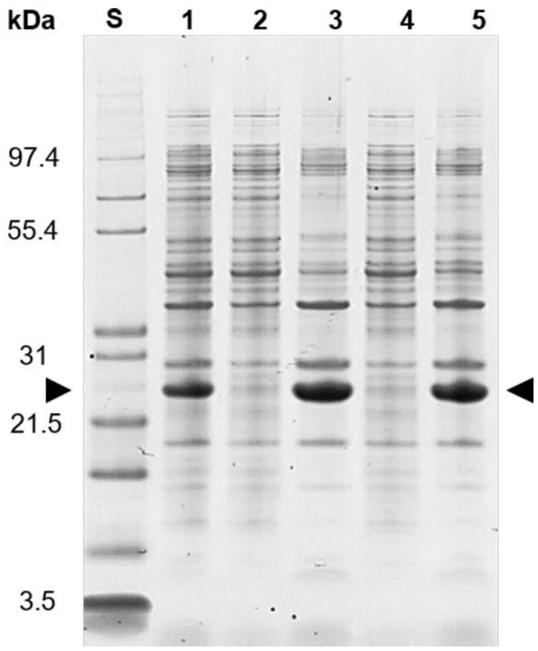

Figure S6 - *Application of 0.4 M ammonium sulfate and 1.5 M sodium chloride on the generated start material containing the target molecule and host cell proteins. An Invitrogen™ Mark12™ Unstained Standard (lane S) was used and the target molecule is indicated by arrows. Molecular weights of selected proteins contained in the standard are shown on the left. The lanes are: start material (lane 1); supernatant (lane 2) and pellet (lane 3) of 0.4M ammonium sulfate precipitation after centrifugation; supernatant (lane 4) and pellet (lane 5) of 1.5M sodium chloride precipitation after centrifugation.*

S7. Buffer compositions and processing temperature of the different purification routes

Table S2 – Summary of the buffer compositions at different processing steps of the performed purification routes regarding their buffer substances, urea content, pH and temperature

Sample	Salts/Buffer	Urea	pH	T
	-	M	-	°C
**Lysate**	20mM Na-PB	0	8	22
**Inclusion body dissolving**	20mM Na-PB	4	8	22
**High-salt precipitation**	20mM Na-PB + 0.4M AMS	4	8	25
**ITC – Cold spin**	-	0	Neutral	4
**ITC – Hot spin**	-	0	Neutral	25
**IMAC – Affinity chromatography**	20mM Na-PB + 0.5 M NaCl + 0.5M imidazole	4	7.4	22
**All processes - Formulation**	20mM Na-PB	4	8	22

S8. Nucleic acid content in the different purification routes

Table S3 - A260/A280 ratios during the performed purification routes. For the high-salt precipitation, the pellet and supernatant were analyzed as well as the final formulation. As the ITC process starts after the HSP only measurements for the cold and hot spin are listed. IMAC feed before application on the chromatography column was compared to the different fractions of the chromatogram (n = 3).

**Start material**	**1.71 ± 0.01**				
**HSP**		**ITC**		**IMAC**	
HSP - Supernatant	1.94 ± 0.01	Cold spin - Supernatant	1.85 ± 0.07	IMAC - Feed	1.75 ± 0.05
HSP - Pellet	0.97 ± 0.04	Cold spin - Pellet	0.86 ± 0.02	IMAC - Flow Through	1.82 ± 0.13
Formulation - Pellet	0.89 ± 0.02	Hot spin - Supernatant	0.52 ± 0.00	IMAC - Column Wash	1.51 ± 0.01
Formulation - Supernatant	0.99 ± 0.01	Hot spin - Pellet	2.15 ± 0.01	IMAC - Eluate	0.46 ± 0.00

S9. Sample load with and without imidazole

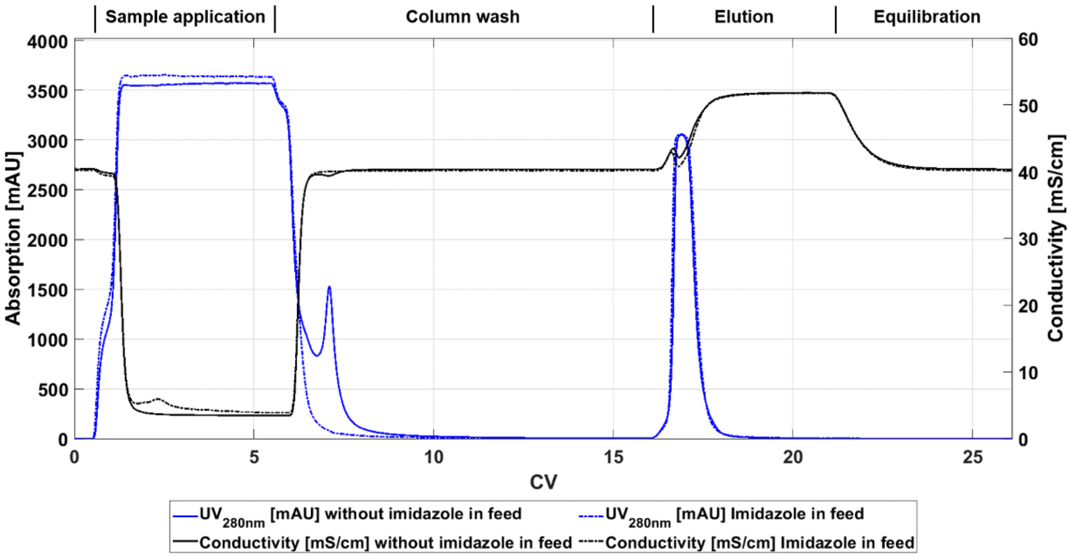

Figure S9 - *Chromatogram of IMAC purification of feed solutions with (line) and without (dashed line) 20mM imidazole. Sample application was performed with a sample pump. Method sections are shown above the graph.*

S10. Size exclusion chromatography

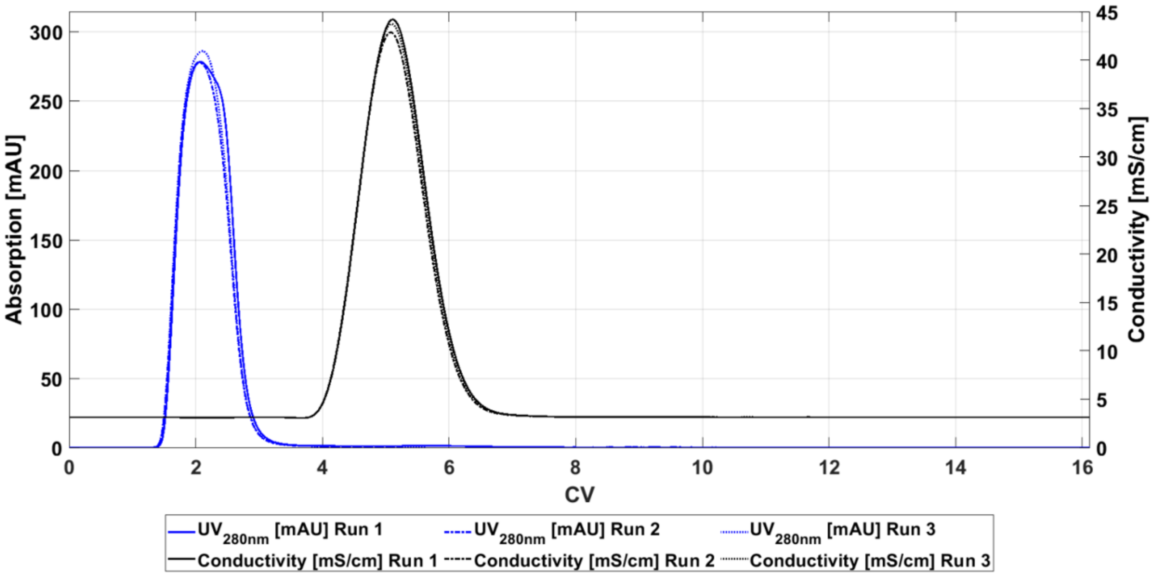

Figure S10 - *Chromatogram of size exclusion chromatography after IMAC purification. Absorbance at 280 nm (blue) indicates a full separation from ions which are correlated to the solution conductivity (black)*

S11. Nickel assay for ELP[V2Y-45]

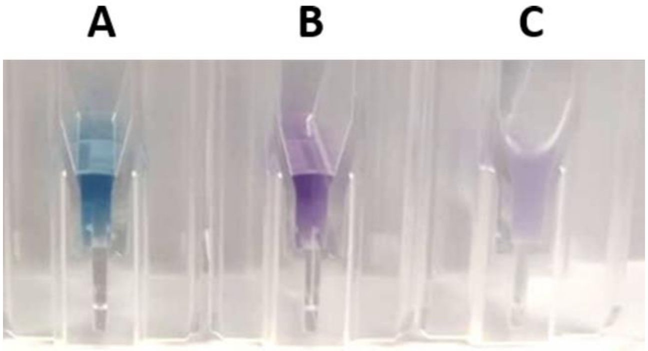

***Figure S11 -** Three examples for Ni^{2+} determination. (A) Assay solution without Ni^{2+}, the blue color is caused by HNB. (B) An IMAC elution fraction without protein load is added to the assay solution, the HNB - Ni^{2+} complex shows purple color. (C) IMAC eluate containing [ELPV2Y-45] was used to perform the Ni^{2+} assay. The combination of HNB and imidazole seems to trigger the phase transition of ELP[V2Y-45], resulting in an increase in turbidity.*
